# Comprehensive Analysis of Copy Number Variations in Kidney Cancer by Single-Cell Exome Sequencing

**DOI:** 10.3389/fgene.2019.01379

**Published:** 2020-01-23

**Authors:** Wenyang Zhou, Fan Yang, Zhaochun Xu, Meng Luo, Pingping Wang, Yu Guo, Huan Nie, Lifen Yao, Qinghua Jiang

**Affiliations:** ^1^School of Life Science and Technology, Harbin Institute of Technology, Harbin, China; ^2^Department of Neurology, The First Affiliated Hospital of Harbin Medical University, Harbin, China

**Keywords:** copy number variations, single-cell exome sequencing, clear-cell renal cell carcinoma, receptor protein tyrosine kinase, signaling transduction pathway

## Abstract

Clear-cell renal cell carcinoma (ccRCC) is the most common and lethal subtype of kidney cancer. *VHL* and *PBRM1* are the top two significantly mutated genes in ccRCC specimens, while the genetic mechanism of the *VHL/PBRM1*-negative ccRCC remains to be elucidated. Here we carried out a comprehensive analysis of single-cell genomic copy number variations (CNVs) in *VHL/PBRM1*-negative ccRCC. Genomic CNVs were identified at the single-cell level, and the tumor cells showed widespread amplification and deletion across the whole genome. Functional enrichment analysis indicated that the amplified genes are significantly enriched in cancer-related signaling transduction pathways. Besides, receptor protein tyrosine kinase (RTK) genes also showed widespread copy number variations in cancer cells. Our studies indicated that the genomic CNVs in RTK genes and downstream signaling transduction pathways may be involved in *VHL/PBRM1*-negative ccRCC pathogenesis and progression, and highlighted the role of the comprehensive investigation of genomic CNVs at the single-cell level in both clarifying pathogenic mechanism and identifying potential therapeutic targets in cancers.

## Introduction

Renal cell carcinoma (RCC) is one kind of kidney cancer, accounting for nearly 300,000 new cancer cases per year worldwide (Hakimi et al., 2013). RCC includes several histological subtypes, among which clear cell renal cell carcinoma (ccRCC) is the most common and lethal one (Hakimi et al., 2016). Increasing studies have shown that the development of ccRCC seems to be shaped by chromosomal lesions and a number of somatic mutations (Sato et al., 2013). *VHL* and *PBRM1*, located within the chromosome 3p25 and 3p21 segments, are the top two significantly mutated genes in ccRCC (Sato et al., 2013). Nearly 90% of ccRCCs undertake the deletion on chromosome 3p, leading to a very high frequency of *VHL* inactivation (Gnarra et al., 1994). Moreover, *VHL* and *PBRM1* are mutated in about 50 and 41% of sporadic ccRCC, respectively (Kaelin, 2004; Varela et al., 2011). However, little is known about the genetic mechanisms in *VHL*/*PBRM1*-negative ccRCC.

Based on the next-generation sequencing technology, previous studies identified many driver mutations in ccRCC (Gnarra et al., 1994; Kaelin, 2004; Sato et al., 2013; Cheng et al., 2019). However, limited insights are available on the genomic diversity within tumor tissues (Wang et al., 2014). Generally, tumor tissues may contain cancer cells from multiple clones and noncancerous cells, which make it difficult to identify the mutations in each clone and detect the driver genes during the cancer progression (Xu et al., 2012; Casasent et al., 2018). Fortunately, single-cell DNA sequencing has been developed to meet this challenge, because it can provide unique insights into intratumor heterogeneity, development, and diversity of cancers at the single-cell level (Casasent et al., 2018). For example, Xu et al. (2012) carried out the single-cell exome sequencing on a ccRCC tumor and its adjacent normal tissue. They identified four genes (i.e., *AHNAK, SRGAP3, LRRK2*, and *USP6*) as potential driving factors for *VHL/PBRM1*-negative ccRCC development, which provided new insights into the pathogenesis of the ccRCC.

Genomic copy number variations (CNVs) play an important role in cancer progression, and emerging studies indicate that genomic CNVs are associated with the ccRCC (Gerlinger et al., 2014; Nouhaud et al., 2018) and other cancers (Waddell et al., 2015; Secrier et al., 2016; Hong et al., 2019). Xu et al. (Xu et al., 2012) performed a single-cell exome sequencing to elucidate the genetic mechanisms of the ccRCC by identifying the single nucleotide variants (SNVs). However, the authors did not examine whether the genomic copy number variations play a crucial role in ccRCC.

To further investigate the potential roles of CNVs in *VHL/PBRM1*-negative ccRCC, we performed a comprehensive single-cell CNV analysis based on a dataset provided by Xu et al., (2012). We delineated the genomic copy number variation landscape at the single-cell level and reclassified all single cells based on the single-cell genomic CNVs. We also identified several significantly amplified/deleted loci and genes in cancer cells. Finally, we further investigated the biological pathways which may be involved in the ccRCC pathogenesis.

## Methods

### Datasets

The sample data and information used in our article came from a previous study, and the original sequencing data were downloaded from NCBI (http://www.ncbi.nlm.nih.gov/sra) under the accession number SRA050201.

### Quality Control

Quality control of the sequencing data was performed using FastQC. The adapter and low-quality ends were trimmed from reads using Trim-Galore version 0.5.0 (http://www.bioinformatics.babraham.ac.uk/projects/trim_galore/). Trimmed reads shorter than 20 bp were discarded.

### Reads Mapping

The human reference genome sequence (Hg19) was used for mapping (http://hgdownload.soe.ucsc.edu/goldenPath/hg19/bigZips/). Short read pairs were mapped to the reference genome using Burrows-Wheeler Aligner (BWA) version 0.7.12-r1039 (Li and Durbin, 2009). In this process, we adopted the BWA-MEM algorithm and adjusted the main parameters, setting the minimum seed length to 19, the penalty for a mismatch to 4, and shorter split hits were marked as secondary. Then, Samtools was used to convert SAM files to compressed BAM files, sort the BAM files by chromosomal coordinates, and remove the PCR duplicates from BAM files.

### Copy Number Variations Calling

In each cell, germline and somatic copy number variations were called by Control-FREEC version 11.5 (Boeva et al., 2012). Considering the exome enrichment during library construction, read counts were calculated by exome region. The target region file of exome capture was downloaded from the Agilent website (https://earray.chem.agilent.com/suredesign/index.htm). The germline CNVs were detected in each cell and bulk normal tissue, respectively. Somatic CNVs were detected only in single cells. Gene annotations were performed with Annovar software (Wang et al., 2010) and OAHG database (Cheng et al., 2016).

### Dimensionality Reduction of Cells

T-distributed stochastic neighbor embedding (t-SNE) was performed based on the germline CNVs of target regions. Both 25 single cells and bulk normal tissue were projected to 2D space using the R package named “Rtsne.”

### Significantly Somatic Copy Number Variation Loci Analysis

Significantly amplified/deleted loci in tumor cells were identified using GISTIC2.0 (Mermel et al., 2011). GISTIC2.0 was run on an input defined as the log_2_()-1 of somatic copy number values, with confidence (-conf) threshold of 0.9. Considering for downstream analysis, *thresholds suggested by GISTIC2.0 for copy number variation were as follows*: if GISTIC score ≥0.9, it means amplification; 0.1 < GISTIC score <0.9, corresponding to gain; −1.3 < GISTIC score < −0.1, loss; GISTIC score ≤ −1.3, deletion.

### Receptor Protein Tyrosine Kinase Gene Copy Number Profiling

To examine the landscape of copy number variations in RTK genes, we derived GISTIC-equivalent scores by dividing the germline copy numbers and classifying genes as amplified if score ≥ 0.9, deleted if score ≤ −1.3, gained if score > 0.1, and loss if the score < −0.1.

### Function Analysis

The significantly amplified and deleted genes were identified according to significantly somatic CNV loci (q-value < 0.0001) in GISTIC2.0. The Kyoto Encyclopedia of Genes and Genomes (KEGG) pathway function enrichment analysis was performed using the Carcinogenic Potency Database (CPDB) (Kamburov et al., 2013). In this study, the p-value threshold for KEGG enrichment analysis is 0.05.

## Results

### Identification of Single-Cell Genomic Copy Number Variations in Kidney Cancer and Normal Cells

To identify genomic CNVs in ccRCC, we analyzed the sequencing data from a ccRCC patient, which includes 20 single-cell exome sequencing data from the tumor tissue, 5 single-cell exome sequencing data from the adjacent normal tissue, and a bulk exome sequencing data from the adjacent normal tissue. Trim-Galore was used to remove the low-quality and adapter segments and analyze the quality of sequencing reads. The cleaned reads were mapped to the reference genome with BWA software (Li and Durbin, 2009). The sequencing depth was more than 20X (29.68 ± 5.68) in all single cells. The genomic CNVs were called by using Control-FREEC (Boeva et al., 2012).

**Figure 1:**
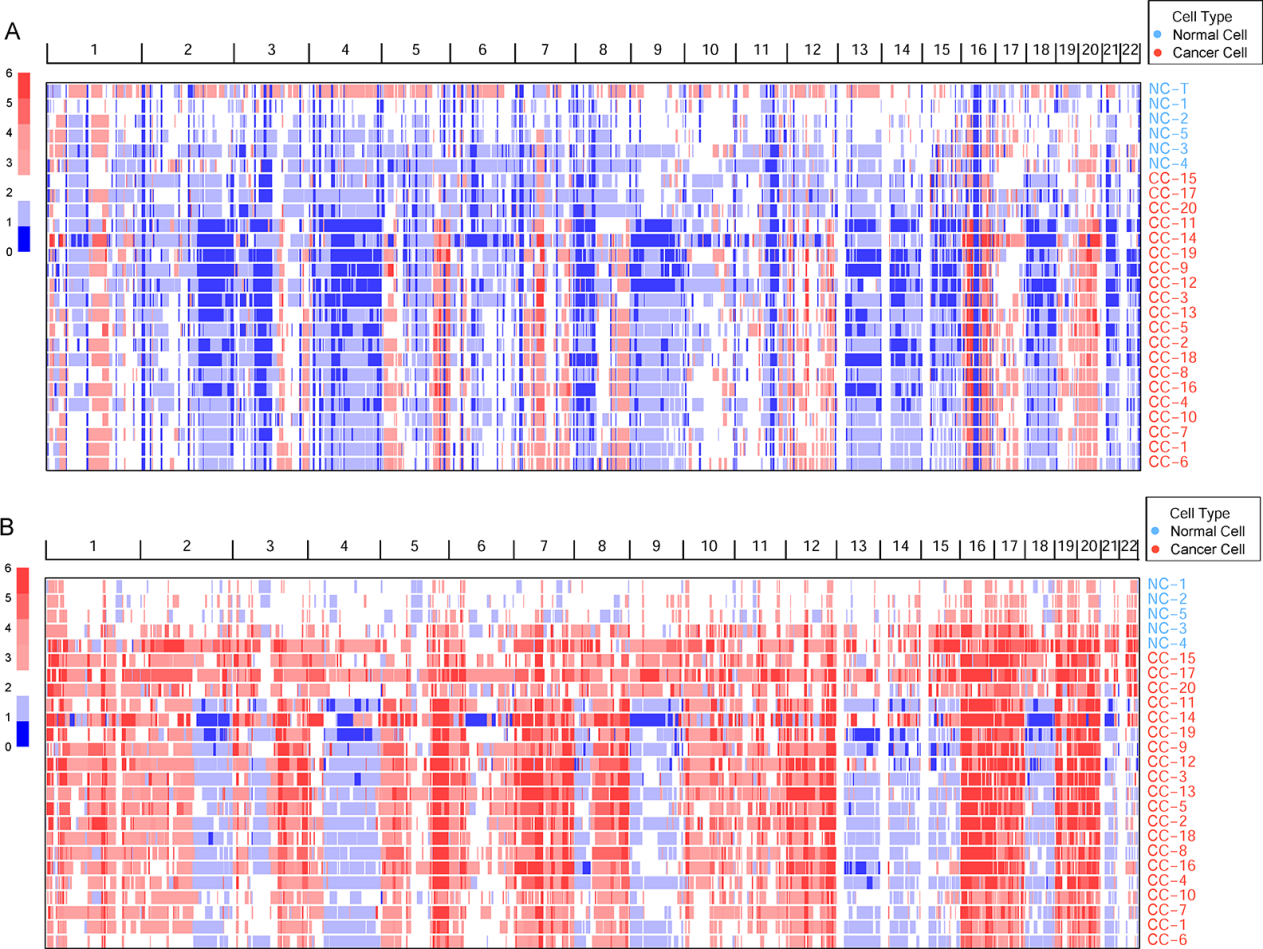
The genomic copy number variations (CNVs) identified across all cells. **(A)** The germline CNVs in single cells and normal tissue. Genomic CNVs within the whole genome are shown, the color scale ranges from blue (deletion) to red (amplification) with estimated copy numbers shown. The cell names are marked by different cell types. **(B)** The somatic CNVs in single cells.

Germline CNVs were identified in all the samples. The comparison between cancer and normal cells revealed widespread amplification and deletion across the whole genome in tumor cells (**Figure 1A**). At the same time, some deleted loci were found both in normal and cancer cells, which may be caused by multiple displacement amplification (MDA) amplification (Yilmaz and Singh, 2012) or exome capture during DNA library preparation.

To remove the background mutations caused by germline or technology flaws, somatic CNVs were identified in all cells using bulk normal tissue as control. The somatic CNVs showed much more amplification than germline CNVs in the cancer cells remarkably (**Figure 1B**). Large-scale of somatic CNVs were found in the ccRCC single cells, which was consistent with the previous studies based on the bulk sequencing (Cancer Genome Atlas Research, N, 2013; Gerlinger et al., 2014; Nouhaud et al., 2018). What’s more, single-cell sequencing data revealed the amplification of copy number showed a high degree of consistency, which suggests the amplification may play an important role in the progression of ccRCC. On the contrary, the deletion showed higher intratumor heterogeneity in the cancer cells.

### Re-Classification of Kidney Cancer and Normal Cells Based on Single-Cell Copy Number Variations

**Figure 2:**
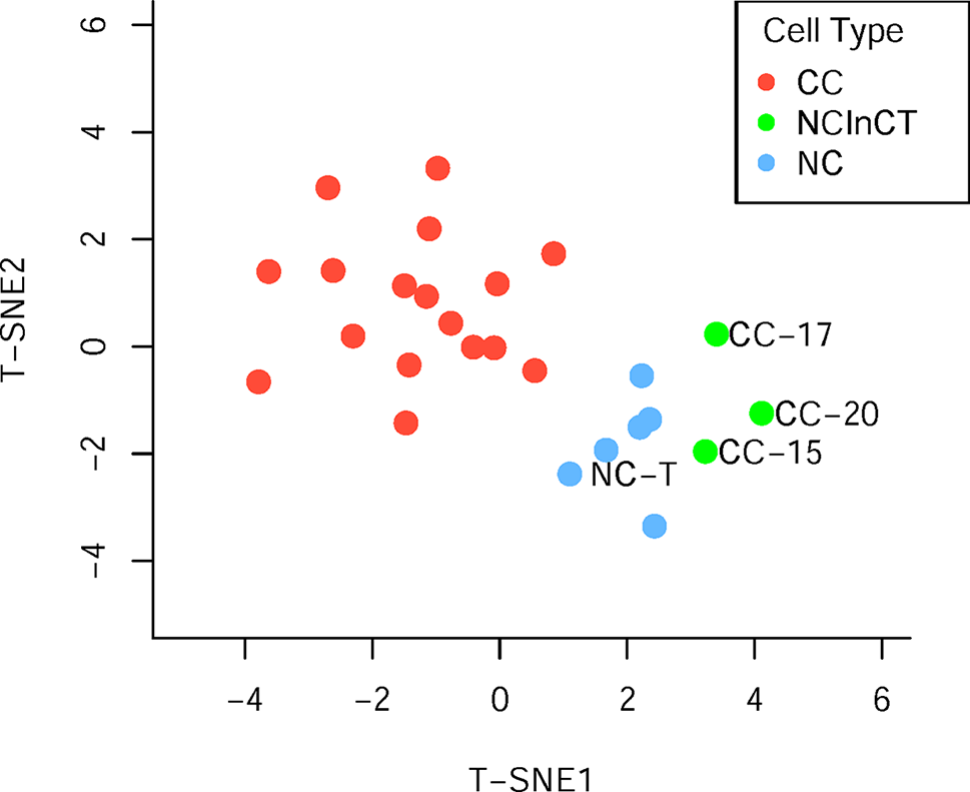
Population analysis based on the germline copy number variations (CNVs). T-distributed stochastic neighbor embedding (T-SNE) analysis of cancer cell (red), normal cell (blue), and normal cell in cancer tissue (green) based on the germline CNVs.

Generally, surgically removed cancer tumors may contain both cancer and normal cells (Xu et al., 2012). To reclassify all the single cells accurately, the t-distributed stochastic neighbor embedding (t-SNE) was performed based on the cell copy number in exome target regions. The results of dimensionality reduction (**Figure 2**, **Supplementary Table S1**) showed that three cancer cells (CC-15, CC-17, and CC-20) clustered tightly with the normal cells and tissue, suggesting that they probably were normal cells in the tumor tissue. These results were consistent with the previous findings which based on the single-cell SNVs (Xu et al., 2012). These three cells (CC-15, CC-17, and CC-20) were excluded from the cancer cell group in the downstream analysis. Focusing on the remaining cancer cells, we found no subpopulation of cancer cells within the cancer tissue.

**Figure 3:**
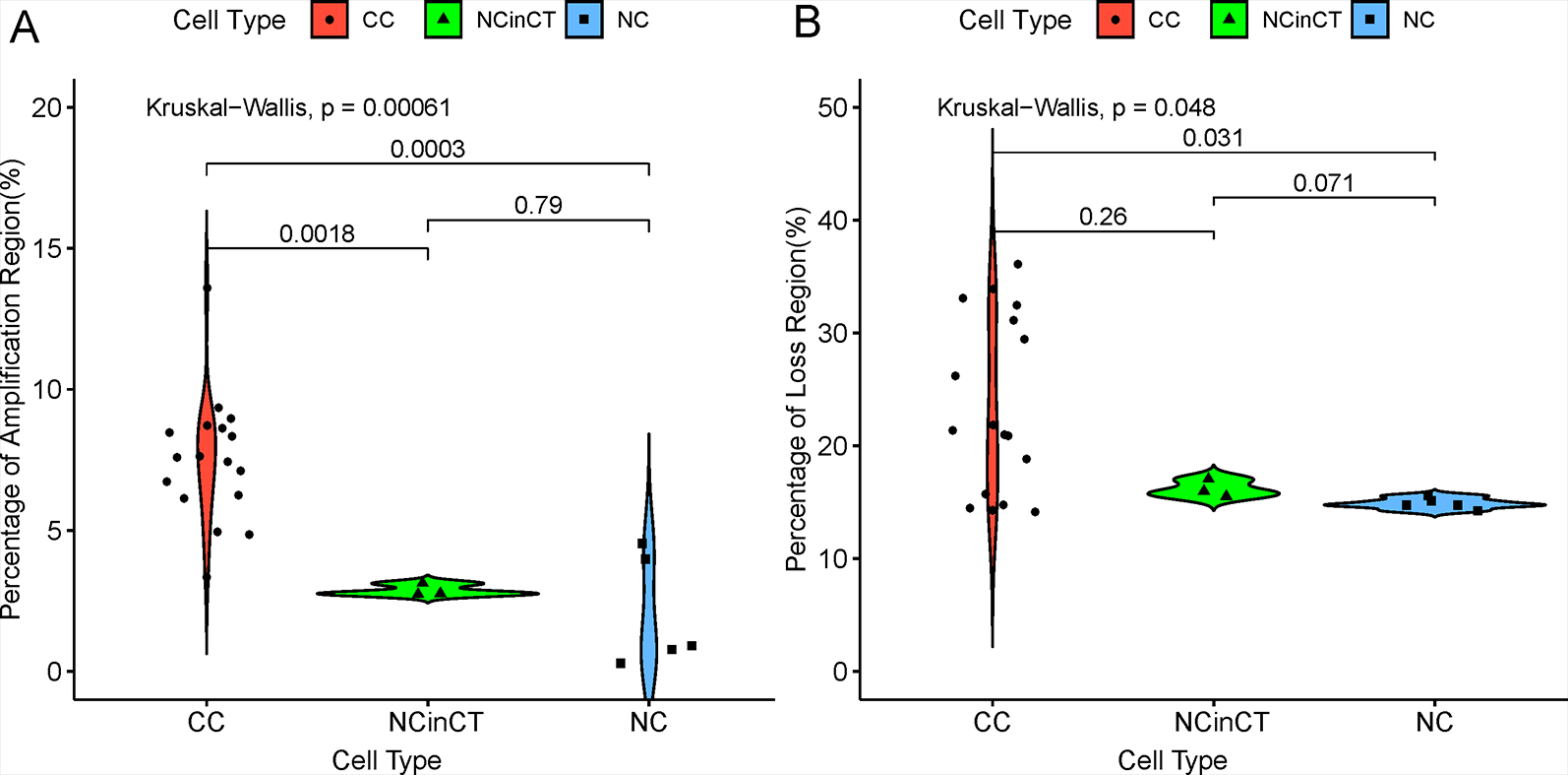
The coverage of genomic copy number variations (CNV) regions in three cell types. **(A)** The percentage of amplification region (copy number ≥ 4) across the whole genome in different cell types. **(B)** The percentage of loss region (copy number = 0) across the whole genome in different cell types. In the two sub-graphs **(A)** and **(B)**, p-values between two groups (Wilcoxon signed-rank test) and all groups (Kruskal-Wallis test) were calculated.

According to the single-cell genomic CNVs, all the single cells can be reclassified into three groups, namely cancer cell (CC), normal cell (NC), and normal cell in cancer tissue (NCinCT). To address whether the genomic CNVs were significantly different between the three groups, we calculated the proportion of whole genome that covered with amplification (copy number ≥ 4) and loss (copy number = 0), respectively. The results (**Figure 3**) showed that there were more amplified loci in CC group than NC group (P = 3×10^−4^) and NCinCT group (P = 1.8×10^−3^). Besides, there was no significant difference between NC and NCinCT groups (P = 0.79). The lost loci also showed a similar result. Single-cell genomic CNVs indicated that the genome of cancer cells was in an extremely unstable state.

### Loci Distribution of Significant Genomic Copy Number Variations in Kidney Cancer

To investigate the loci distribution of the significant genomic CNVs across all tumor single cells, GISTIC2.0 (Mermel et al., 2011) was used to identify the significant genomic CNVs loci based on the somatic CNVs in 17 cancer cells, but not including germline CNVs which are not involved in cancer development generally. The results indicated that copy numbers in the significant CNV loci have a high degree of consistency across all the cancer cells. Although lots of lost loci (more slight than deletion, −1.3 < GISTIC score < −0.1) were identified, there was no significantly deleted locus (GISTIC score ≤ −1.3) found in cancer cells, which was consistent with high heterogeneity of deletion region in our cancer cells.

**Figure 4:**
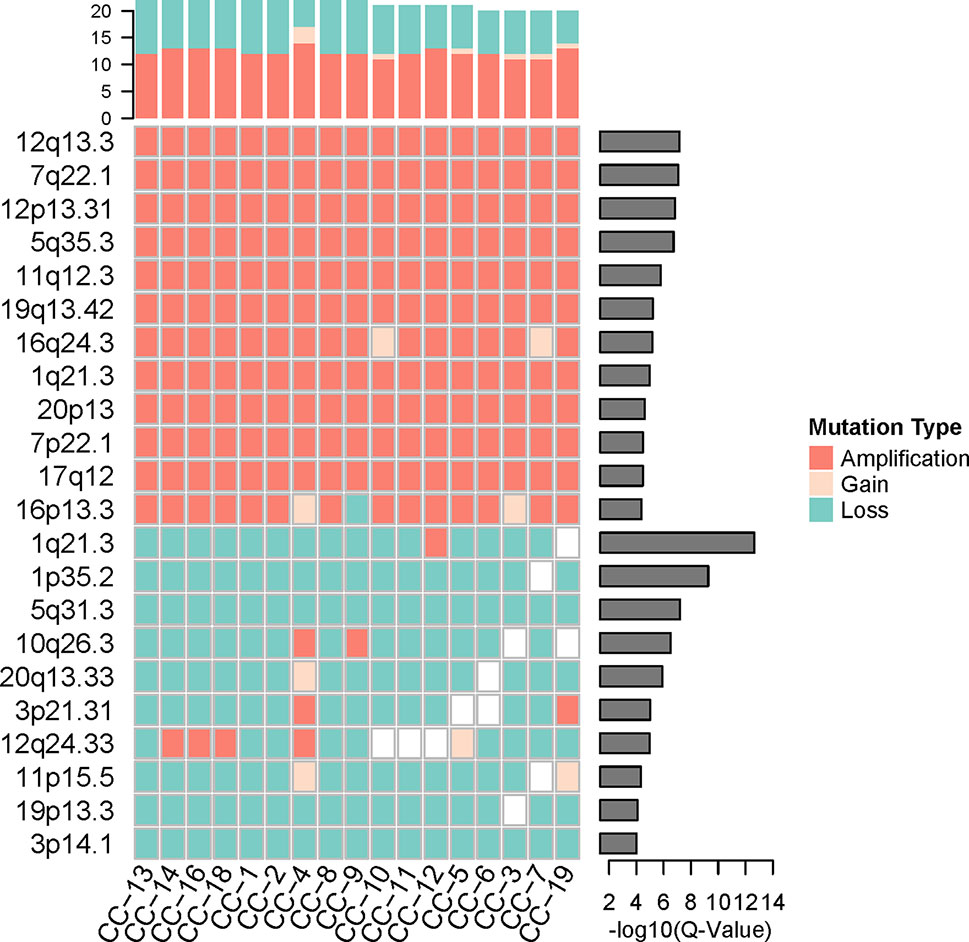
The significant genomic copy number variation (CNV) loci in cancer cells. All CNV types in each cancer cell were counted for the top frequency histogram, and q-value for each significant genomic CNV loci was shown on the right. Only the loci with q-value < 0.0001 were shown.

Significantly amplified loci (**Figure 4**, **Supplementary Table S2A**) according to GISTIC2.0 (12q13.3, 12p13.31, 5q35.3, etc.; q-value < 0.05) comprised genes such as *IGFBP4*, *ERBB2*, *ERBB3*, *FGFR4*, *CDK2*, *FLT4*, and so on. The *IGFBP4* gene had been reported to be associated with several types of cancer (Hallberg et al., 2000; Romero et al., 2011; Yang et al., 2017), it can promote the RCC cell metastasis and activate Wnt/beta-catenin signaling pathway in humans (Ueno et al., 2011). *ERBB2* and *ERBB3* genes belong to the epidermal growth factor receptor (*EGFR*) family, and they had been identified as common driver genes of multiple cancer types by promoting solid tumor growth (Yarden, 2001; Henson et al., 2017; Oldrini et al., 2017). The amplification of *EGFR* also was found in other cancers, which contributed to the *EGFR* excessive activation (Sigismund et al., 2018). *FGFR4* gene belongs to the fibroblast growth factor receptor family, and the activation of *FGFR4* can promote cell growth and angiogenesis in cancer (Bai et al., 2015). *CDK2* gene is commonly excessive activation in human cancers, and dysfunction of *CDK2* can lead to uncontrolled cell growth (Mihara et al., 2001). *FLT4* gene, belonging to the vascular endothelial growth factor family, had been reported to regulate cancer cell survival and proliferation (Varney and Singh, 2015).

While the top significantly deleted loci (**Figure 4**, **Supplementary Table S2B**) (1q21.3, 1p35.2, 16q24.3, 3p14.1, etc.; q-value < 0.05) showed loss of *Chmp1A*, *CADM2*, *PRAP1*, and *ULK1* genes. *Chmp1A* and *CADM2*, belonging to cell adhesion molecules family, had been found to be a tumor suppressor gene in RCC. The overexpression of *Chmp1A* and *CADM2* significantly suppressed cancer growth and invasion (You et al., 2012; He et al., 2013). *PARP1* gene played an important role in DNA repair and cell apoptosis (Tulin, 2011), the cell with *PARP1* deficiency show resistance to DNA damage-induced programmed cell death and increased cancer risk (Schiewer and Knudsen, 2014). *ULK1* was an initiate autophagy gene, and the down-regulation of *ULK1* had been found in cancer (Zhang et al., 2017). *ULK1* may play a pivotal role in cancer by promoting cell death (Chen et al., 2014).

The genes in significantly amplified loci include a number of known driver genes, which may promote the cancer progression by the up-regulation of cell growth and cell cycle. Significantly deleted loci include some tumor suppressor genes and autophagy genes. The inactivation of these genes leads to uncontrolled tumor growth, which may contribute to the *VHL/PBRM1*-negative ccRCC pathogenesis and progression

### Functional Analysis of Significant Genomic Copy Number Variations in Kidney Cancer

**Figure 5:**
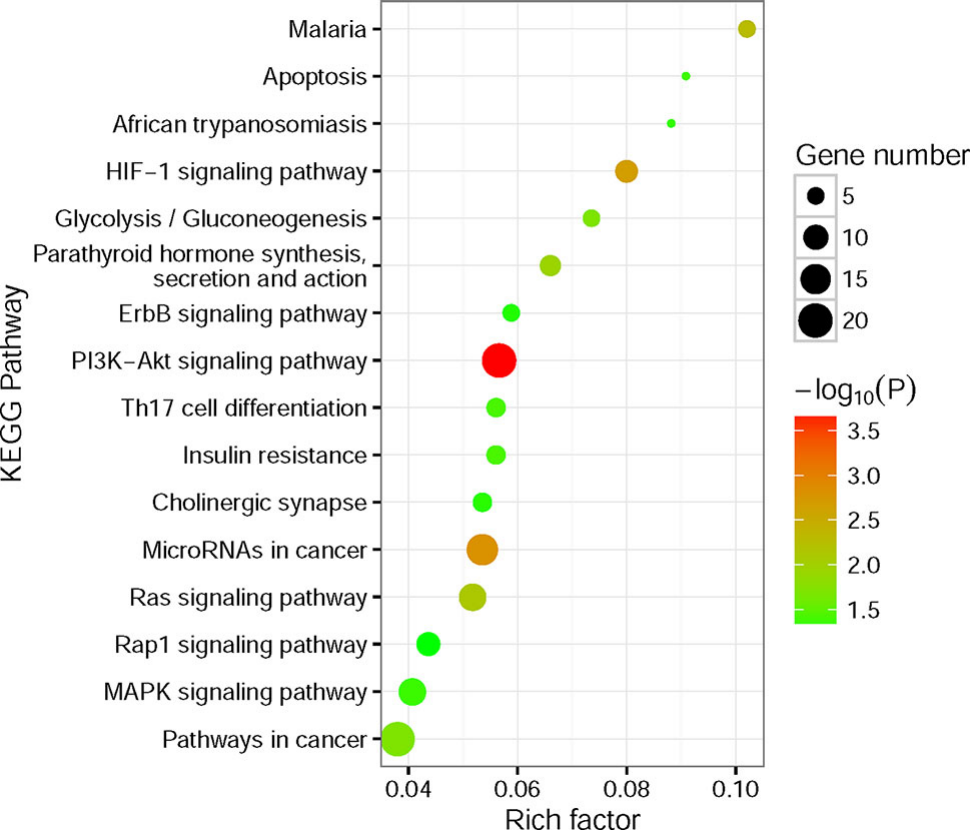
Kyoto Encyclopedia of Genes and Genomes (KEGG) functional enrichment analysis for significantly amplified genes. The size of the point means the gene number both in our amplified gene set and KEGG pathway terms. The color of point means enrichment significance (−log_10_P). The pathways were sorted by rich factor (the ratio of significantly amplified gene number in this pathway term to gene number in this pathway term).

To better understand the potential biological and functional characteristics of the significantly amplified and deleted genes in cancer cells, biological function pathways in ccRCC had been further investigated. The KEGG functional enrichment analysis was performed using the CPDB Database based on the amplified and deleted genes, respectively. The amplified genes showed significant enrichment (p-value < 0.05) for signal transduction, metabolism, cell cycle, immunity, and other cancer-related pathways (**Figure 5**, **Supplementary Table S3**). In contrast to amplified genes, deleted genes only showed significant enrichment for the fatty acid elongation pathway (p-value = 7.6×10^−3^).

The most notable result is that a large portion of enrichment pathways belong to the signaling transduction pathway. Both of the HIF-1 (Posadas et al., 2013), ErbB (Liu et al., 2015), PI3K-Akt (Linehan et al., 2010; Sato et al., 2013; Guo et al., 2015), Ras (de Araujo Junior et al., 2015; Chen et al., 2018), Rap1 (Chen et al., 2018), and MAPK signaling pathway (Liu et al., 2015) had been found involved in the pathogenesis of RCC. What’s more, the results also showed that Th17 cells (Li et al., 2015) and microRNAs (Gowrishankar et al., 2014) seem to have a connection with the ccRCC pathogenesis. Interestingly, the fatty acid elongation pathway was significantly deleted in ccRCC, which may account for the fact that ccRCC tumors are lipid-laden (Hakimi et al., 2016).

### Receptor Protein Tyrosine Kinase Genes Show Widespread Copy Number Variations in Cancer Cells

Since lots of cancer-related signaling transduction pathways showed significantly amplified in cancer cells, we then examined the copy number variations in their upstream RTK genes (Robinson et al., 2000; Secrier et al., 2016) to investigate possible reasons for the negative results that tumor did not appear known driver mutations in *VHL* and *PBRM1*.

**Figure 6:**
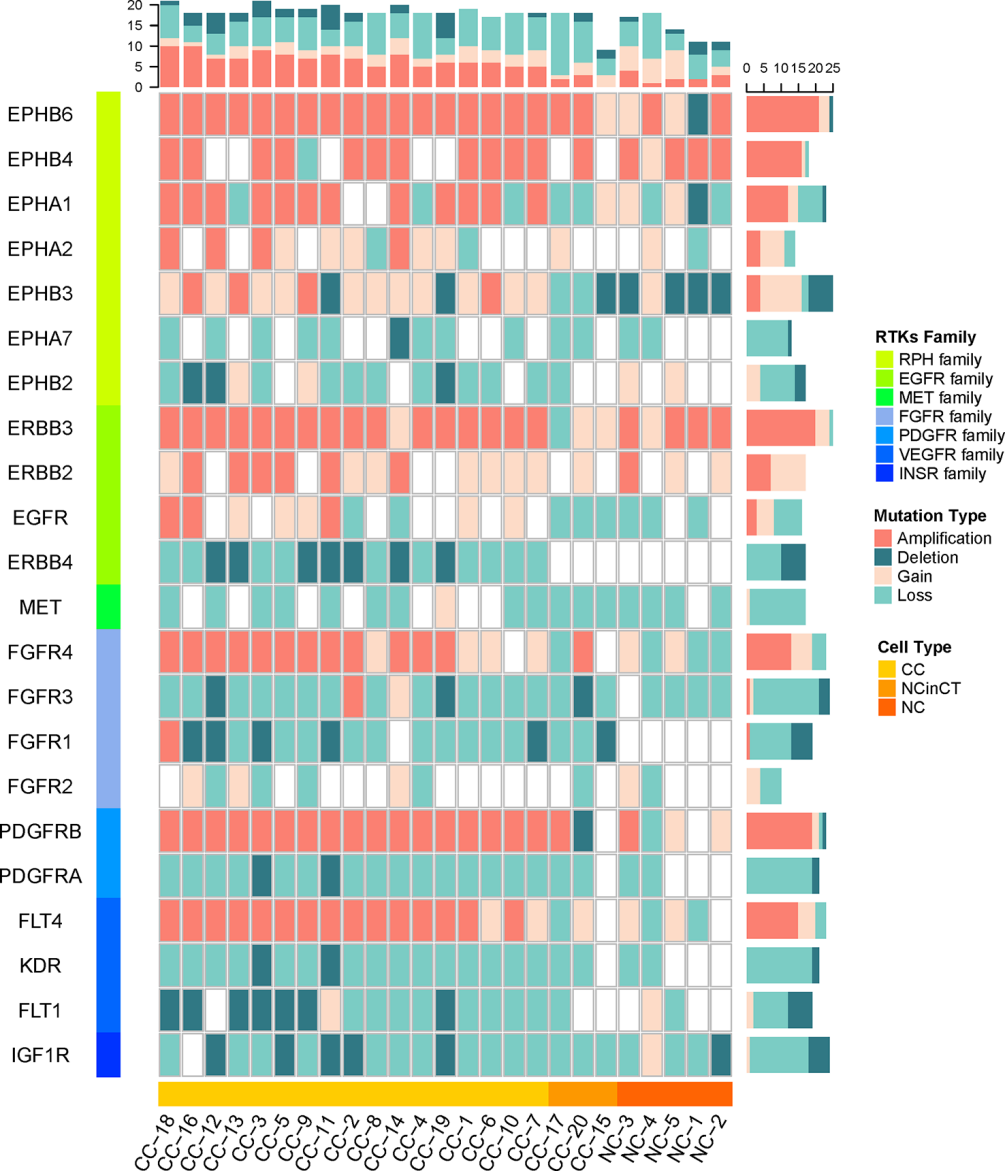
The copy number of receptor protein tyrosine kinase (RTK) genes in all single cells. The copy number variations (CNVs) on RTK genes in both tumor and normal cells were shown. The RTKs family and cell types were shown at the left and bottom of the plot. The mutation types in each cell and gene were counted for the top and right frequency histograms, respectively.

The single cancer cells show widespread amplification and deletion on multiple RTKs compared with the normal cells, the NC and NCinCT groups show similar RTK gene profile. There were some RTK genes (*EPHB6*, *EPHA1*, *EPHB3*, *FGFR4*, *PDGFRB*, and *FLT4*) showing amplification in cancer cells. On the contrary, *EPHB2*, *ERBB4*, *FGFR1*, *PDGFRA*, *KDR*, and *FLT1* genes showed deletion in cancer cells (**Figure 6**). Genomic copy number is varied across these RTKs and downstream pathways, indicating that the genomic CNVs in RTKs and downstream signaling transduction pathways may have important roles in the pathogenesis and progression of the *VHL/PBRM1*-negative ccRCC.

## Discussion

Previous studies have shown that *VHL* and *PBRM1* are the top two significantly mutated genes in ccRCC. However, the pathogenesis in *VHL/PBRM1*-negative ccRCC is still unclear. Our comprehensive analysis of CNVs in 25 single cells from a ccRCC patient provided new insights into the pathogenesis of the ccRCC. We reclassified all the single cells and identified pathological mutations in *VHL/PBRM1*-negative ccRCC cells. Similar to the genomic CNVs in other cancers, the pathogenesis in *VHL/PBRM1*-negative ccRCC seems to be shaped by the accumulation of amplification in driver genes (*IGFBP4*, *ERBB2*, *ERBB3*, *FGFR4*, *CDK2*, and *FLT4*), the loss of function in tumor suppressor genes (*Chmp1A*, *CADM2*) and autophagy genes (*PRAP1*, *ULK1*).

Pathway analysis of these significantly amplified and deleted genes identified several signaling transduction pathways, including HIF-1, ErbB, PI3K-Akt, Ras, Rap1, and MAPK signaling pathways, were affected by genomic amplification. At the same time, RTK genes showed widespread copy number variations in cancer cells specifically. Mutations on RTKs may take part in the overactivity of downstream signaling transduction pathways, leading to the uncontrolled growth of ccRCC cells.

Overall, our single-cell analysis of the copy number in *VHL/PBRM1*-negative ccRCC revealed that the genomic CNVs in RTKs may cooperate with downstream signaling transduction pathways to take part in *VHL/PBRM1*-negative ccRCC pathogenesis and progression. Clinically, our findings may provide more effective targeted therapeutic approaches for patients with *VHL*/*PBRM1*-negative ccRCC. However, because of the small number of cells and the high intratumor heterogeneity, our findings need to be verified in larger cohorts.

## Data Availability Statement

Publicly available datasets were analyzed in this study. The original sequencing data can be downloaded from NCBI (http://www.ncbi.nlm.nih.gov/sra) under the accession number SRA050201.

## Author Contributions

HN, LY, and QJ designed the experiments. PW obtained data from NCBI. WZ and ML analyzed the data. FY, ZX, and YG wrote the manuscript. All authors read and approved the manuscript.

## Funding

This work is supported by the National Natural Science Foundation of China [61571152, 61822108], the National Science and Technology Major Project of China [2016YFC1202302] and the Natural Science Foundation of Heilongjiang Province [F2015006].

## Conflict of Interest

The authors declare that the research was conducted in the absence of any commercial or financial relationships that could be construed as a potential conflict of interest.

## Supplementary Material

Table S1 The results of dimensionality reduction based on the germline CNVs. The name and coordinate in 2D space of all single cells were shown in this table.

Table S2 The results of significantly amplified (**Table S2A**) and deleted (**Table S2B**) loci according to GISTIC2.0. The cytoband name, q-value and gene names of each amplification/deletion loci were shown in this table.

Table S3 The results of KEGG enrichment analysis based on significantly amplified (**Table S3A**) and deleted (**Table S3B**) genes according to the CPDB database. The pathway name, p-value and gene sets of each pathway were shown in this table.
